# 2-Hydr­oxy-3-nitro-*N*-phenyl­benzamide

**DOI:** 10.1107/S1600536809013063

**Published:** 2009-04-18

**Authors:** Abdul Rauf Raza, Muhammad Danish, M. Nawaz Tahir, Bushra Nisar, Gyungse Park

**Affiliations:** a Department of Chemistry, University of Sargodha, Sargodha, Pakistan; b Department of Physics, University of Sargodha, Sargodha, Pakistan; c Department of Chemistry, Kunsan National University, Kusan, Chonbuk 573-701, Republic of Korea

## Abstract

The asymmetric unit of the title compound, C_13_H_10_N_2_O_4_, contains two crystallographically independent mol­ecules. The aromatic rings are oriented at dihedral angles of 24.39 (3) and 7.47 (3)° in the two mol­ecules and intra­molecular N—H⋯O and O—H⋯O hydrogen bonds result in the formation of two planar six-membered rings. In the crystal structure, inter­molecular O—H⋯O and C—H⋯O hydrogen bonds link the mol­ecules into chains, forming *R*_2_^2^(10) ring motifs. Weak π–π contacts between the benzene and phenyl rings [centroid–centroid distance = 3.955 (3) Å] may further stabilize the structure.

## Related literature

For general background to the biological activity of benzoxazepine derivatives, see: Clark *et al.* (2006 ▶); Mc Gee *et al.* (2001 ▶). For a related structure, see: Yi *et al.* (2007 ▶). For ring-motifs, see: Bernstein *et al.* (1995 ▶).
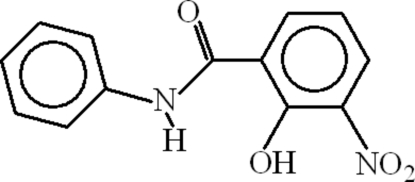


## Experimental

### 

#### Crystal data

C_13_H_10_N_2_O_4_
*M* *_r_* = 258.23
Monoclinic, 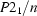

*a* = 10.485 (2) Å
*b* = 11.465 (2) Å
*c* = 20.013 (4) Å
β = 101.181 (5)°
*V* = 2360.1 (8) Å^3^
*Z* = 8
Mo *K*α radiation
μ = 0.11 mm^−1^
*T* = 296 K
0.26 × 0.20 × 0.18 mm

#### Data collection

Bruker Kappa APEXII CCD area-detector diffractometer
Absorption correction: multi-scan (*SADABS*; Bruker, 2005 ▶) *T*_min_ = 0.979, *T*_max_ = 0.986
13479 measured reflections
4190 independent reflections
1880 reflections with *I* > 2σ(*I*)
*R*_int_ = 0.091

#### Refinement

*R*[*F* ^2^ > 2σ(*F* ^2^)] = 0.048
*wR*(*F* ^2^) = 0.097
*S* = 0.88
4190 reflections
339 parameters
H-atom parameters constrained
Δρ_max_ = 0.19 e Å^−3^
Δρ_min_ = −0.19 e Å^−3^

### 

Data collection: *APEX2* (Bruker, 2007 ▶); cell refinement: *SAINT* (Bruker, 2007 ▶); data reduction: *SAINT*; program(s) used to solve structure: *SHELXS97* (Sheldrick, 2008 ▶); program(s) used to refine structure: *SHELXL97* (Sheldrick, 2008 ▶); molecular graphics: *ORTEP-3 for Windows* (Farrugia, 1997 ▶) and *PLATON* (Spek, 2009 ▶); software used to prepare material for publication: *WinGX* publication routines (Farrugia, 1999 ▶) and *PLATON*.

## Supplementary Material

Crystal structure: contains datablocks global, I. DOI: 10.1107/S1600536809013063/hk2662sup1.cif

Structure factors: contains datablocks I. DOI: 10.1107/S1600536809013063/hk2662Isup2.hkl

Additional supplementary materials:  crystallographic information; 3D view; checkCIF report

## Figures and Tables

**Table 1:** Hydrogen-bond geometry (Å, °)

*D*—H⋯*A*	*D*—H	H⋯*A*	*D*⋯*A*	*D*—H⋯*A*
N1—H1*N*⋯O2	0.86	1.93	2.653 (3)	140
O2—H2*O*⋯O3	0.82	1.86	2.569 (3)	144
O2—H2*O*⋯O7^i^	0.82	2.31	2.920 (3)	132
N3—H3*N*⋯O6	0.86	1.95	2.673 (3)	140
O6—H6*O*⋯O7	0.82	1.81	2.532 (3)	146
C6—H6⋯O1^ii^	0.93	2.55	3.231 (5)	131
C18—H18⋯O4^iii^	0.93	2.49	3.167 (4)	130

Symmetry codes: (i) 
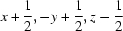
; (ii) 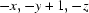; (iii) 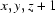.

**Fig. 1.**
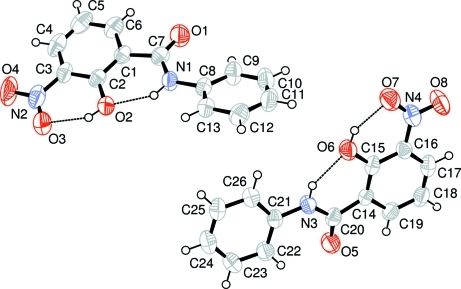
The molecular structure of the title molecule, with the atom-numbering scheme. Displacement ellipsoids are drawn at the 50% probability level. Hydrogen bonds are shown as dotted lines.

**Fig. 2.**
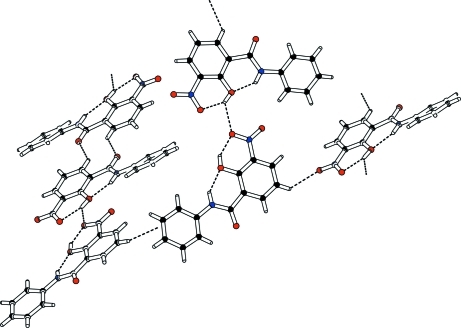
A partial packing diagram of the title compound. Hydrogen bonds are shown as dashed lines.

## supplementary crystallographic information

### Comment

Different biological activities have been reported for benzoxazepine derivatives such as anti-cancer (Mc Gee *et al.*, 2001) and anti-HIV agents (Clark *et al.*,  2006). Our work was aiming the formations of various benzoxazepine derivatives. The title compound, (I), is an intermediate for their preparation. It will also be utilized for the complexation with various metals. We report herein the crystal structure of (I).

The crystal structure of N-(2-Methoxyphenyl) 2-hydroxy-5-methyl-3-nitro- benzamide, (II) (Yi *et al.*,  2007) has been published, which is different from (I) due to the substitution of methoxy on N-phenyl and methyl group on the nitro containing aromatic ring.

The asymmetric unit of (I) contains two crystallographically independent molecules. Rings A (C1-C6), B (C8-C13) and A' (C14-C19), B' (C21-C26) are, of course, planar, and they are oriented at dihedral angles of A/B = 24.39 (3) and A'/B' = 7.47 (3) °. Intramolecular N-H···O and O-H···O hydrogen bonds (Table 1) result in the formations of planar six-membered rings C (O2/N1/C1/C2/C7/H7N), D (O2/O3/N2/C2/C3/H2O) and C' (O6/N3/C14/C15/C20/H3N), D' (O6/O7/N4/C15/C16/H6O). They are oriented with respect to the adjacent rings at dihedral angles of A/C = 3.33 (3), A/D = 0.99 (3), C/D = 2.74 (3) ° and A'/C' = 1.93 (3), A'/D' = 2.71 (3), C'/D' = 3.83 (3) °. So, they are nearly coplanar.

In the crystal structure, intermolecular O-H···O and C-H···O hydrogen bonds (Table 1) link the molecules into chains forming R_2_^2^(10) ring motifs (Fig. 2) (Bernstein *et al.*, 1995), in which they may be effective in the stabilization of the structure. The π–π contact between the benzene and phenyl rings, Cg1—Cg4^i^ [symmetry code: (i) x - 1/2, 1/2 - y, z - 1/2, where Cg1 and Cg4 are centroids of the rings A (C1-C6) and B' (C21-C26), respectively] may further stabilize the structure, with centroid-centroid distance of 3.955 (3) Å.

### Experimental

For the preparation of the title compound, a solution of N-phenyl 2-hydroxy- benzamide (5.3 g, 0.025 mol) in ethylacetate (EtOAc) (25 ml) was added dropwise to a nitrating mixture of HNO_3_ (2.25 ml, 3.15 g, 0.05 mol) and H_2_SO_4_ (1.33 ml, 2.45 g, 0.025 mol), with constant stirring, while the temperature was kept below 278 K. The reaction mixture was refluxed for 5 h, cooled, neutralized with aqueous NaHCO_3_ (10%) and extracted with EtOAc (3 × 25 ml). The organic layer was combined, dried over anhydrous Na_2_SO_4_, filtered and concentrated under reduced pressure to afford reddish brown solid. The column chromatographic purification with 0, 2.5, 5, 7.5 and 10 % EtOAc in petrol (0.5 l each) over a silica gel packed column (25.5 cm) afforded the title compound.

### Refinement

H atoms were positioned geometrically, with O-H = 0.82 Å (for OH), N-H = 0.86 Å (for NH) and C-H = 0.93 Å for aromatic H, and constrained to ride on their parent atoms, with U_iso_(H) = 1.2U_eq_(C,N,O).

### Crystal data

**Table:**

C_13_H_10_N_2_O_4_	*F*(000) = 1072
*M**_r_* = 258.23	*D*_x_ = 1.454 Mg m^−^^3^
Monoclinic, *P*2_1_/*n*	Mo *K*α radiation, λ = 0.71073 Å
Hall symbol: -P 2yn	Cell parameters from 2234 reflections
*a* = 10.485 (2) Å	θ = 2.1–27.0°
*b* = 11.465 (2) Å	µ = 0.11 mm^−^^1^
*c* = 20.013 (4) Å	*T* = 296 K
β = 101.181 (5)°	Prism, brown
*V* = 2360.1 (8) Å^3^	0.26 × 0.20 × 0.18 mm
*Z* = 8	

### Data collection

**Table:**

Bruker Kappa APEXII CCD area-detector diffractometer	4190 independent reflections
Radiation source: fine-focus sealed tube	1880 reflections with *I* > 2σ(*I*)
graphite	*R*_int_ = 0.091
Detector resolution: 7.82 pixels mm^-1^	θ_max_ = 25.1°, θ_min_ = 2.1°
ω scans	*h* = −11→12
Absorption correction: multi-scan (*SADABS*; Bruker, 2005)	*k* = −13→13
*T*_min_ = 0.979, *T*_max_ = 0.986	*l* = −23→22
13479 measured reflections	

### Refinement

**Table:**

Refinement on *F*^2^	Primary atom site location: structure-invariant direct methods
Least-squares matrix: full	Secondary atom site location: difference Fourier map
*R*[*F*^2^ > 2σ(*F*^2^)] = 0.048	Hydrogen site location: inferred from neighbouring sites
*wR*(*F*^2^) = 0.097	H-atom parameters constrained
*S* = 0.88	*w* = 1/[σ^2^(*F*_o_^2^) + (0.0245*P*)^2^] where *P* = (*F*_o_^2^ + 2*F*_c_^2^)/3
4190 reflections	(Δ/σ)_max_ < 0.001
339 parameters	Δρ_max_ = 0.19 e Å^−^^3^
0 restraints	Δρ_min_ = −0.19 e Å^−^^3^

### Special details

**Table:**

Geometry. Bond distances, angles *etc*. have been calculated using the rounded fractional coordinates. All su's are estimated from the variances of the (full) variance-covariance matrix. The cell e.s.d.'s are taken into account in the estimation of distances, angles and torsion angles
Refinement. Refinement of *F*^2^ against ALL reflections. The weighted *R*-factor *wR* and goodness of fit *S* are based on *F*^2^, conventional *R*-factors *R* are based on *F*, with *F* set to zero for negative *F*^2^. The threshold expression of *F*^2^ > σ(*F*^2^) is used only for calculating *R*-factors(gt) *etc*. and is not relevant to the choice of reflections for refinement. *R*-factors based on *F*^2^ are statistically about twice as large as those based on *F*, and *R*- factors based on ALL data will be even larger.

### Fractional atomic coordinates and isotropic or equivalent isotropic displacement parameters (Å^2^)

**Table:**

	*x*	*y*	*z*	*U*_iso_*/*U*_eq_	
O1	0.1388 (2)	0.41856 (19)	0.08311 (10)	0.0808 (10)	
O2	0.4704 (2)	0.34896 (17)	0.00581 (9)	0.0586 (8)	
O3	0.5836 (3)	0.3805 (2)	−0.09569 (12)	0.0815 (11)	
O4	0.4835 (3)	0.4655 (2)	−0.18661 (11)	0.1105 (13)	
O5	0.79454 (13)	0.47437 (11)	0.53123 (6)	0.0742 (10)	
O6	0.44496 (13)	0.29222 (11)	0.47736 (6)	0.0630 (9)	
O7	0.2306 (2)	0.24638 (18)	0.51192 (12)	0.0785 (10)	
O8	0.1932 (2)	0.29716 (17)	0.61025 (11)	0.0891 (11)	
N1	0.3303 (3)	0.32209 (18)	0.10180 (11)	0.0516 (10)	
N2	0.4853 (4)	0.4287 (3)	−0.12935 (14)	0.0728 (14)	
N3	0.6742 (2)	0.35952 (17)	0.45098 (10)	0.0505 (10)	
N4	0.2654 (3)	0.2946 (2)	0.56899 (14)	0.0634 (11)	
C1	0.2574 (3)	0.4220 (2)	−0.00668 (14)	0.0488 (11)	
C2	0.3693 (3)	0.4033 (2)	−0.03317 (14)	0.0475 (11)	
C3	0.3707 (4)	0.4438 (2)	−0.09925 (15)	0.0543 (13)	
C4	0.2652 (4)	0.4996 (3)	−0.13803 (16)	0.0701 (16)	
C5	0.1553 (4)	0.5164 (3)	−0.11226 (16)	0.0768 (16)	
C6	0.1533 (4)	0.4784 (3)	−0.04686 (15)	0.0665 (14)	
C7	0.2363 (4)	0.3878 (3)	0.06361 (15)	0.0541 (14)	
C8	0.3309 (3)	0.2730 (2)	0.16716 (13)	0.0470 (11)	
C9	0.2525 (3)	0.3112 (2)	0.21062 (14)	0.0650 (14)	
C10	0.2579 (4)	0.2549 (3)	0.27241 (16)	0.0726 (16)	
C11	0.3406 (4)	0.1640 (3)	0.29130 (15)	0.0707 (16)	
C12	0.4202 (3)	0.1278 (3)	0.24855 (14)	0.0670 (14)	
C13	0.41554 (13)	0.18195 (11)	0.18641 (6)	0.0553 (11)	
C14	0.5958 (3)	0.4037 (2)	0.55541 (13)	0.0446 (11)	
C15	0.4774 (3)	0.3463 (2)	0.53838 (13)	0.0480 (13)	
C16	0.3931 (3)	0.3471 (2)	0.58522 (15)	0.0485 (11)	
C17	0.4279 (4)	0.4007 (3)	0.64807 (14)	0.0599 (14)	
C18	0.5448 (3)	0.4560 (3)	0.66462 (14)	0.0617 (13)	
C19	0.6269 (3)	0.4582 (2)	0.61847 (13)	0.0543 (11)	
C20	0.6977 (3)	0.4154 (2)	0.51198 (14)	0.0509 (13)	
C21	0.7531 (3)	0.3568 (2)	0.40071 (14)	0.0450 (11)	
C22	0.8800 (3)	0.3951 (2)	0.41066 (15)	0.0574 (14)	
C23	0.9484 (3)	0.3838 (3)	0.35836 (16)	0.0659 (14)	
C24	0.8919 (4)	0.3353 (3)	0.29665 (17)	0.0684 (14)	
C25	0.7655 (4)	0.2979 (2)	0.28709 (15)	0.0614 (14)	
C26	0.6952 (3)	0.3085 (2)	0.33859 (14)	0.0531 (12)	
H1N	0.39778	0.30839	0.08451	0.0619*	
H2O	0.52842	0.34004	−0.01592	0.0704*	
H4	0.26902	0.52571	−0.18160	0.0839*	
H5	0.08309	0.55283	−0.13819	0.0923*	
H6	0.07877	0.49133	−0.02915	0.0798*	
H9	0.19654	0.37395	0.19859	0.0781*	
H10	0.20405	0.27973	0.30148	0.0873*	
H11	0.34297	0.12685	0.33283	0.0848*	
H12	0.47775	0.06650	0.26144	0.0804*	
H13	0.46965	0.15678	0.15758	0.0665*	
H3N	0.60268	0.32091	0.44160	0.0606*	
H6O	0.36959	0.26924	0.47197	0.0756*	
H17	0.37186	0.39896	0.67883	0.0716*	
H18	0.56902	0.49214	0.70679	0.0737*	
H19	0.70571	0.49738	0.62998	0.0653*	
H22	0.91923	0.42808	0.45201	0.0686*	
H23	1.03403	0.40950	0.36497	0.0792*	
H24	0.93892	0.32807	0.26197	0.0820*	
H25	0.72657	0.26515	0.24563	0.0736*	
H26	0.60934	0.28330	0.33162	0.0640*	

### Atomic displacement parameters (Å^2^)

**Table:**

	*U*^11^	*U*^22^	*U*^33^	*U*^12^	*U*^13^	*U*^23^
O1	0.0677 (19)	0.1119 (19)	0.0649 (15)	0.0324 (14)	0.0181 (15)	0.0166 (13)
O2	0.0552 (16)	0.0776 (15)	0.0443 (12)	0.0043 (12)	0.0126 (12)	0.0041 (11)
O3	0.084 (2)	0.107 (2)	0.0586 (16)	−0.0113 (16)	0.0263 (16)	−0.0001 (14)
O4	0.148 (3)	0.145 (2)	0.0445 (14)	−0.0237 (18)	0.0338 (16)	0.0192 (14)
O5	0.069 (2)	0.0960 (18)	0.0604 (14)	−0.0269 (14)	0.0194 (14)	−0.0208 (12)
O6	0.0651 (17)	0.0760 (15)	0.0511 (13)	−0.0161 (12)	0.0194 (12)	−0.0120 (11)
O7	0.078 (2)	0.0900 (18)	0.0719 (16)	−0.0262 (14)	0.0252 (15)	−0.0096 (13)
O8	0.089 (2)	0.1042 (19)	0.0886 (17)	−0.0140 (14)	0.0535 (17)	−0.0023 (14)
N1	0.057 (2)	0.0570 (16)	0.0432 (14)	0.0072 (14)	0.0157 (14)	0.0078 (12)
N2	0.100 (3)	0.075 (2)	0.0454 (19)	−0.0332 (19)	0.019 (2)	−0.0099 (16)
N3	0.0519 (19)	0.0548 (16)	0.0476 (15)	−0.0079 (13)	0.0164 (14)	−0.0049 (12)
N4	0.071 (2)	0.0595 (18)	0.0653 (19)	−0.0036 (16)	0.027 (2)	0.0070 (15)
C1	0.059 (2)	0.0429 (19)	0.0423 (18)	0.0015 (16)	0.0042 (19)	0.0017 (14)
C2	0.058 (2)	0.0405 (18)	0.0409 (18)	−0.0071 (16)	0.0018 (18)	−0.0011 (14)
C3	0.077 (3)	0.048 (2)	0.0386 (18)	−0.0155 (18)	0.013 (2)	−0.0043 (15)
C4	0.106 (4)	0.057 (2)	0.042 (2)	−0.007 (2)	0.001 (2)	0.0049 (16)
C5	0.095 (4)	0.067 (2)	0.058 (2)	0.011 (2)	−0.011 (2)	0.0044 (18)
C6	0.080 (3)	0.062 (2)	0.053 (2)	0.008 (2)	0.002 (2)	0.0008 (17)
C7	0.060 (3)	0.050 (2)	0.050 (2)	−0.0012 (18)	0.005 (2)	−0.0027 (16)
C8	0.055 (2)	0.0468 (18)	0.0386 (17)	−0.0034 (16)	0.0074 (17)	0.0000 (14)
C9	0.084 (3)	0.062 (2)	0.053 (2)	0.0128 (18)	0.023 (2)	0.0026 (17)
C10	0.082 (3)	0.087 (3)	0.056 (2)	0.000 (2)	0.031 (2)	−0.0017 (19)
C11	0.086 (3)	0.081 (3)	0.046 (2)	−0.013 (2)	0.015 (2)	0.0073 (18)
C12	0.073 (3)	0.069 (2)	0.056 (2)	0.0068 (19)	0.005 (2)	0.0142 (17)
C13	0.058 (2)	0.064 (2)	0.0445 (18)	0.0009 (18)	0.0116 (18)	0.0024 (16)
C14	0.050 (2)	0.0461 (18)	0.0366 (17)	0.0028 (15)	0.0055 (17)	0.0040 (14)
C15	0.064 (3)	0.0441 (19)	0.0368 (17)	0.0061 (17)	0.0120 (18)	0.0041 (14)
C16	0.051 (2)	0.047 (2)	0.0494 (19)	0.0040 (16)	0.0145 (19)	0.0091 (15)
C17	0.079 (3)	0.060 (2)	0.045 (2)	0.011 (2)	0.023 (2)	0.0081 (16)
C18	0.074 (3)	0.072 (2)	0.0388 (18)	0.009 (2)	0.010 (2)	−0.0004 (16)
C19	0.056 (2)	0.061 (2)	0.0437 (18)	0.0045 (16)	0.0046 (18)	0.0021 (15)
C20	0.061 (3)	0.050 (2)	0.0416 (18)	0.0078 (17)	0.0099 (18)	0.0059 (15)
C21	0.055 (2)	0.0384 (17)	0.0440 (18)	0.0040 (16)	0.0159 (18)	0.0034 (14)
C22	0.058 (3)	0.062 (2)	0.0540 (19)	−0.0056 (18)	0.015 (2)	−0.0025 (16)
C23	0.057 (3)	0.074 (2)	0.073 (2)	−0.0056 (19)	0.028 (2)	0.0035 (19)
C24	0.081 (3)	0.071 (2)	0.062 (2)	0.006 (2)	0.036 (2)	0.0071 (19)
C25	0.079 (3)	0.062 (2)	0.0467 (19)	0.0059 (19)	0.021 (2)	0.0014 (16)
C26	0.058 (2)	0.051 (2)	0.053 (2)	−0.0010 (16)	0.0175 (19)	−0.0003 (15)

### Geometric parameters (Å, °)

**Table:**

O1—C7	1.215 (5)	C12—C13	1.382 (3)
O2—C2	1.342 (3)	C4—H4	0.9300
O3—N2	1.246 (5)	C5—H5	0.9300
O4—N2	1.218 (4)	C6—H6	0.9300
O2—H2O	0.8200	C9—H9	0.9300
O5—C20	1.219 (3)	C10—H10	0.9300
O6—C15	1.353 (3)	C11—H11	0.9300
O7—N4	1.258 (4)	C12—H12	0.9300
O8—N4	1.224 (4)	C13—H13	0.9300
O6—H6O	0.8200	C14—C19	1.389 (4)
N1—C8	1.423 (3)	C14—C20	1.508 (4)
N1—C7	1.353 (4)	C14—C15	1.388 (4)
N2—C3	1.455 (6)	C15—C16	1.408 (4)
N1—H1N	0.8600	C16—C17	1.384 (4)
N3—C21	1.421 (4)	C17—C18	1.363 (5)
N3—C20	1.358 (3)	C18—C19	1.380 (4)
N4—C16	1.446 (4)	C21—C26	1.389 (4)
N3—H3N	0.8600	C21—C22	1.378 (4)
C1—C2	1.395 (4)	C22—C23	1.385 (4)
C1—C6	1.384 (5)	C23—C24	1.379 (5)
C1—C7	1.517 (4)	C24—C25	1.371 (6)
C2—C3	1.404 (4)	C25—C26	1.384 (5)
C3—C4	1.379 (5)	C17—H17	0.9300
C4—C5	1.364 (6)	C18—H18	0.9300
C5—C6	1.384 (4)	C19—H19	0.9300
C8—C9	1.379 (4)	C22—H22	0.9300
C8—C13	1.376 (3)	C23—H23	0.9300
C9—C10	1.386 (4)	C24—H24	0.9300
C10—C11	1.362 (5)	C25—H25	0.9300
C11—C12	1.370 (5)	C26—H26	0.9300
			
O1···C9	2.876 (3)	C13···C19^iv^	3.463 (3)
O1···C5^i^	3.332 (5)	C14···C15^x^	3.431 (3)
O1···C6^i^	3.231 (5)	C14···C14^x^	3.479 (4)
O2···N2	2.888 (3)	C15···C20^x^	3.337 (4)
O2···O3	2.569 (3)	C15···C14^x^	3.431 (3)
O2···N1	2.653 (3)	C16···C20^x^	3.374 (4)
O2···C3^ii^	3.273 (4)	C17···C21^x^	3.401 (4)
O2···O7^iii^	2.920 (3)	C17···O4^xii^	3.330 (4)
O2···C22^iv^	3.414 (3)	C17···N3^x^	3.437 (4)
O2···C2^ii^	3.292 (3)	C18···O4^xii^	3.167 (4)
O3···C1^ii^	3.283 (4)	C19···C8^xi^	3.427 (4)
O3···O7^iii^	2.800 (3)	C19···C13^xi^	3.463 (3)
O3···O2	2.569 (3)	C20···C16^x^	3.374 (4)
O3···C23^iv^	3.396 (4)	C20···C15^x^	3.337 (4)
O3···C7^ii^	3.250 (5)	C20···N1^xi^	3.407 (3)
O4···C17^v^	3.330 (4)	C21···C17^x^	3.401 (4)
O4···C18^v^	3.167 (4)	C21···C2^xi^	3.392 (4)
O5···C22	2.879 (3)	C22···O5	2.879 (3)
O6···O7	2.532 (3)	C22···O2^xi^	3.414 (3)
O6···N4	2.872 (3)	C23···O3^xi^	3.396 (4)
O6···N3	2.673 (3)	C24···N2^xi^	3.428 (5)
O7···O2^vi^	2.920 (3)	C26···C3^xi^	3.523 (4)
O7···O3^vi^	2.800 (3)	C2···H1N	2.5600
O7···C6^vii^	3.329 (4)	C5···H12^iv^	2.9900
O7···C5^vii^	3.389 (4)	C7···H9	2.8200
O7···O6	2.532 (3)	C11···H26	3.1000
O1···H9	2.3300	C12···H26	2.9300
O1···H6^i^	2.5500	C13···H19^iv^	3.0600
O1···H5^i^	2.7800	C15···H3N	2.5600
O1···H6	2.3600	C20···H22	2.8200
O1···H11^viii^	2.9100	C24···H4^ii^	3.0300
O2···H1N	1.9300	C25···H4^ii^	2.9000
O3···H2O	1.8600	H1N···O2	1.9300
O4···H17^v^	2.8300	H1N···H13	2.3000
O4···H18^v^	2.4900	H1N···C2	2.5600
O4···H4	2.3700	H2O···O3	1.8600
O5···H23^ix^	2.8100	H2O···O7^iii^	2.3100
O5···H22	2.3000	H2O···N2	2.4500
O5···H19	2.3600	H3N···H26	2.2600
O6···H3N	1.9500	H3N···C15	2.5600
O7···H2O^vi^	2.3100	H3N···O6	1.9500
O7···H6O	1.8100	H4···C25^ii^	2.9000
O8···H13^vi^	2.7400	H4···O4	2.3700
O8···H17	2.4000	H4···C24^ii^	3.0300
O8···H25^vi^	2.7600	H5···H12^iv^	2.5000
N1···C20^iv^	3.407 (3)	H5···O1^i^	2.7800
N1···O2	2.653 (3)	H6···H6^i^	2.2100
N2···O2	2.888 (3)	H6···O1^i^	2.5500
N2···C24^iv^	3.428 (5)	H6···O1	2.3600
N3···C17^x^	3.437 (4)	H6O···O7	1.8100
N3···C1^xi^	3.407 (3)	H6O···N4	2.4200
N3···O6	2.673 (3)	H9···C7	2.8200
N4···O6	2.872 (3)	H9···O1	2.3300
N4···C5^vii^	3.367 (4)	H11···O1^vii^	2.9100
N2···H2O	2.4500	H12···C5^xi^	2.9900
N4···H6O	2.4200	H12···H5^xi^	2.5000
C1···N3^iv^	3.407 (3)	H13···H1N	2.3000
C1···O3^ii^	3.283 (4)	H13···O8^iii^	2.7400
C2···C2^ii^	3.575 (4)	H17···O4^xii^	2.8300
C2···C21^iv^	3.392 (4)	H17···O8	2.4000
C2···O2^ii^	3.292 (3)	H18···O4^xii^	2.4900
C3···C26^iv^	3.523 (4)	H19···O5	2.3600
C3···O2^ii^	3.273 (4)	H19···C13^xi^	3.0600
C5···O7^viii^	3.389 (4)	H22···O5	2.3000
C5···N4^viii^	3.367 (4)	H22···C20	2.8200
C5···O1^i^	3.332 (5)	H23···O5^ix^	2.8100
C6···O7^viii^	3.329 (4)	H25···O8^iii^	2.7600
C6···O1^i^	3.231 (5)	H26···C11	3.1000
C7···O3^ii^	3.250 (5)	H26···C12	2.9300
C8···C19^iv^	3.427 (4)	H26···H3N	2.2600
C9···O1	2.876 (3)		
			
C2—O2—H2O	109.00	C11—C10—H10	119.00
C15—O6—H6O	109.00	C12—C11—H11	120.00
C7—N1—C8	127.8 (3)	C10—C11—H11	120.00
O3—N2—O4	121.5 (4)	C13—C12—H12	120.00
O4—N2—C3	119.0 (3)	C11—C12—H12	120.00
O3—N2—C3	119.5 (3)	C8—C13—H13	120.00
C7—N1—H1N	116.00	C12—C13—H13	120.00
C8—N1—H1N	116.00	C15—C14—C19	118.5 (3)
C20—N3—C21	128.1 (2)	C15—C14—C20	126.9 (2)
O7—N4—O8	121.0 (3)	C19—C14—C20	114.6 (3)
O8—N4—C16	120.4 (3)	O6—C15—C16	121.6 (3)
O7—N4—C16	118.7 (3)	O6—C15—C14	119.7 (2)
C21—N3—H3N	116.00	C14—C15—C16	118.7 (2)
C20—N3—H3N	116.00	N4—C16—C15	121.4 (3)
C2—C1—C7	126.7 (3)	N4—C16—C17	117.3 (3)
C2—C1—C6	118.5 (3)	C15—C16—C17	121.3 (3)
C6—C1—C7	114.8 (3)	C16—C17—C18	119.6 (3)
C1—C2—C3	118.1 (3)	C17—C18—C19	119.6 (3)
O2—C2—C3	123.4 (3)	C14—C19—C18	122.2 (3)
O2—C2—C1	118.6 (2)	O5—C20—N3	122.7 (3)
N2—C3—C4	117.3 (3)	O5—C20—C14	120.5 (2)
C2—C3—C4	122.0 (4)	N3—C20—C14	116.8 (2)
N2—C3—C2	120.7 (3)	N3—C21—C22	124.7 (2)
C3—C4—C5	119.8 (3)	N3—C21—C26	115.4 (3)
C4—C5—C6	118.8 (3)	C22—C21—C26	119.9 (3)
C1—C6—C5	122.8 (4)	C21—C22—C23	119.2 (3)
N1—C7—C1	116.5 (3)	C22—C23—C24	121.3 (3)
O1—C7—N1	123.1 (3)	C23—C24—C25	119.1 (3)
O1—C7—C1	120.4 (3)	C24—C25—C26	120.6 (3)
N1—C8—C13	116.4 (2)	C21—C26—C25	119.9 (3)
N1—C8—C9	123.9 (2)	C16—C17—H17	120.00
C9—C8—C13	119.7 (2)	C18—C17—H17	120.00
C8—C9—C10	119.2 (3)	C17—C18—H18	120.00
C9—C10—C11	121.2 (3)	C19—C18—H18	120.00
C10—C11—C12	119.4 (3)	C14—C19—H19	119.00
C11—C12—C13	120.4 (3)	C18—C19—H19	119.00
C8—C13—C12	120.1 (2)	C21—C22—H22	120.00
C3—C4—H4	120.00	C23—C22—H22	120.00
C5—C4—H4	120.00	C22—C23—H23	119.00
C6—C5—H5	121.00	C24—C23—H23	119.00
C4—C5—H5	121.00	C23—C24—H24	120.00
C5—C6—H6	119.00	C25—C24—H24	120.00
C1—C6—H6	119.00	C24—C25—H25	120.00
C10—C9—H9	120.00	C26—C25—H25	120.00
C8—C9—H9	120.00	C21—C26—H26	120.00
C9—C10—H10	119.00	C25—C26—H26	120.00
			
C8—N1—C7—O1	3.6 (5)	C13—C8—C9—C10	−1.7 (4)
C8—N1—C7—C1	−176.0 (2)	N1—C8—C13—C12	−178.7 (2)
C7—N1—C8—C9	−19.2 (4)	C9—C8—C13—C12	1.1 (4)
C7—N1—C8—C13	160.6 (3)	N1—C8—C9—C10	178.0 (3)
O3—N2—C3—C2	−0.8 (4)	C8—C9—C10—C11	1.1 (5)
O3—N2—C3—C4	178.8 (3)	C9—C10—C11—C12	0.2 (5)
O4—N2—C3—C2	−179.6 (3)	C10—C11—C12—C13	−0.8 (5)
O4—N2—C3—C4	0.1 (4)	C11—C12—C13—C8	0.2 (4)
C20—N3—C21—C26	−169.9 (2)	C19—C14—C15—O6	−179.8 (2)
C21—N3—C20—O5	1.0 (4)	C19—C14—C15—C16	0.9 (4)
C21—N3—C20—C14	179.7 (2)	C20—C14—C15—O6	0.8 (4)
C20—N3—C21—C22	11.9 (4)	C20—C14—C15—C16	−178.5 (2)
O8—N4—C16—C15	−179.4 (2)	C15—C14—C19—C18	0.7 (4)
O8—N4—C16—C17	−0.9 (4)	C20—C14—C19—C18	−179.9 (3)
O7—N4—C16—C17	179.2 (3)	C15—C14—C20—O5	175.5 (2)
O7—N4—C16—C15	0.8 (4)	C15—C14—C20—N3	−3.3 (4)
C7—C1—C2—C3	−178.7 (3)	C19—C14—C20—O5	−3.9 (3)
C6—C1—C7—N1	173.5 (3)	C19—C14—C20—N3	177.4 (2)
C6—C1—C2—C3	0.6 (4)	O6—C15—C16—N4	−2.8 (4)
C2—C1—C6—C5	0.4 (5)	O6—C15—C16—C17	178.8 (2)
C7—C1—C6—C5	179.7 (3)	C14—C15—C16—N4	176.5 (2)
C2—C1—C7—O1	173.1 (3)	C14—C15—C16—C17	−1.9 (4)
C2—C1—C7—N1	−7.3 (4)	N4—C16—C17—C18	−177.1 (3)
C6—C1—C7—O1	−6.2 (4)	C15—C16—C17—C18	1.4 (5)
C6—C1—C2—O2	179.8 (3)	C16—C17—C18—C19	0.2 (5)
C7—C1—C2—O2	0.5 (4)	C17—C18—C19—C14	−1.2 (5)
O2—C2—C3—N2	−0.2 (4)	N3—C21—C22—C23	177.6 (3)
O2—C2—C3—C4	−179.9 (3)	C26—C21—C22—C23	−0.5 (4)
C1—C2—C3—N2	179.0 (3)	N3—C21—C26—C25	−177.6 (2)
C1—C2—C3—C4	−0.7 (4)	C22—C21—C26—C25	0.6 (4)
N2—C3—C4—C5	−179.8 (3)	C21—C22—C23—C24	0.1 (5)
C2—C3—C4—C5	−0.2 (5)	C22—C23—C24—C25	0.1 (5)
C3—C4—C5—C6	1.1 (5)	C23—C24—C25—C26	0.0 (5)
C4—C5—C6—C1	−1.2 (5)	C24—C25—C26—C21	−0.4 (4)

Symmetry codes: (i) −*x*, −*y*+1, −*z*; (ii) −*x*+1, −*y*+1, −*z*; (iii) *x*+1/2, −*y*+1/2, *z*−1/2; (iv) *x*−1/2, −*y*+1/2, *z*−1/2; (v) *x*, *y*, *z*−1; (vi) *x*−1/2, −*y*+1/2, *z*+1/2; (vii) −*x*+1/2, *y*−1/2, −*z*+1/2; (viii) −*x*+1/2, *y*+1/2, −*z*+1/2; (ix) −*x*+2, −*y*+1, −*z*+1; (x) −*x*+1, −*y*+1, −*z*+1; (xi) *x*+1/2, −*y*+1/2, *z*+1/2; (xii) *x*, *y*, *z*+1.

### Hydrogen-bond geometry (Å, °)

**Table:**

*D*—H···*A*	*D*—H	H···*A*	*D*···*A*	*D*—H···*A*
N1—H1N···O2	0.86	1.93	2.653 (3)	140
O2—H2O···O3	0.82	1.86	2.569 (3)	144
O2—H2O···O7^iii^	0.82	2.31	2.920 (3)	132
N3—H3N···O6	0.86	1.95	2.673 (3)	140
O6—H6O···O7	0.82	1.81	2.532 (3)	146
C6—H6···O1^i^	0.93	2.55	3.231 (5)	131
C18—H18···O4^xii^	0.93	2.49	3.167 (4)	130

Symmetry codes: (iii) *x*+1/2, −*y*+1/2, *z*−1/2; (i) −*x*, −*y*+1, −*z*; (xii) *x*, *y*, *z*+1.

## References

[bb1] Bernstein, J., Davis, R. E., Shimoni, L. & Chang, N.-L. (1995). *Angew. Chem. Int. Ed. Engl.* **34**, 1555–1573.

[bb2] Bruker (2005). *SADABS* Bruker AXS Inc. Madison, Wisconsin, USA.

[bb3] Bruker (2007). *APEX2* and *SAINT* Bruker AXS Inc., Madison, Wisconsin, USA.

[bb4] Clark, D., Dedova, I., Cordwell, S. & Matsumoto, I. (2006). *J. Mol. Psychiatr.* **11**, 459–470. 10.1038/sj.mp.4001806 16491132

[bb5] Farrugia, L. J. (1997). *J. Appl. Cryst.* **30**, 565.

[bb6] Farrugia, L. J. (1999). *J. Appl. Cryst.* **32**, 837–838.

[bb7] Mc Gee, M. M., Campiani, G., Ramunno, A., Fattorusso, C., Nacci, V., Lawler, M., Williams, D. C. & Zisterer, D. M. (2001). *J. Pharmacol. Exp. Ther.* **296**, 31–40. 11123359

[bb8] Sheldrick, G. M. (2008). *Acta Cryst.* A**64**, 112–122. 10.1107/S0108767307043930 18156677

[bb9] Spek, A. L. (2009). *Acta Cryst.* D**65**, 148–155. 10.1107/S090744490804362X PMC2631630 19171970

[bb10] Yi, H.-P., Wu, J., Ding, K.-L., Jiang, X.-K. & Li, Z.-T. (2007). *J. Org. Chem.* **72**, 870–877. 10.1021/jo0619940 17253806

